# Effects of the COVID-19 Pandemic on Orthodontic Practices in Europe: Findings From a Survey by the European Federation of Orthodontics

**DOI:** 10.7759/cureus.104730

**Published:** 2026-03-05

**Authors:** Miltiadis A Makrygiannakis, Angel Alonso Tosso, Eleftherios G Kaklamanos

**Affiliations:** 1 School of Dentistry, National and Kapodistrian University of Athens, Athens, GRC; 2 School of Dentistry, European University Cyprus, Nicosia, CYP; 3 Orthodontics, Private Clinic, Cadiz, ESP; 4 School of Dentistry, Aristotle University of Thessaloniki, Thessaloniki, GRC; 5 Dentistry, Hamdan Bin Mohammed College of Dental Medicine, Mohammed Bin Rashid University of Medicine and Health Sciences, Dubai, ARE

**Keywords:** covid-19, europe, feo, orthodontics and dentofacial orthopedics, survey research

## Abstract

Background: The COVID-19 pandemic created major disruptions for healthcare systems globally, with dentistry, especially orthodontics, facing challenges due to the nature of close-contact treatments and procedures that generate aerosols. This research, conducted by the European Federation of Orthodontics (FEO), aimed to assess the pandemic's impact on orthodontic practices across Europe and to identify the clinical, financial, and operational adjustments made during this period.

Methodology: An online cross-sectional survey was conducted among orthodontists who are members of national societies associated with the FEO from June to September 2022. The questionnaire gathered demographic information and insights on clinical adaptations, infection control measures, patient management, and financial implications. Descriptive and inferential statistics were used to assess the correlations between responses and variables, including gender, country, years of experience, practice type, and vaccination status.

Results: A total of 428 orthodontists from various European countries and countries with national orthodontic associations affiliated with the FEO participated in the survey. The majority observed significant changes in their clinical operations, such as lengthier appointments, the implementation of triage protocols, and an increased reliance on PPE and ventilation strategies. While teleorthodontics was broadly embraced, many practitioners discontinued its use after the lockdown. Financially, most respondents reported experiencing revenue losses and a lack of government support. Stress levels differed across regions, with Greece and the UK showing the highest levels. Treatment delays and compromises in outcomes occurred more frequently in countries that enforced stricter lockdowns. Private practitioners recovered more swiftly than those working in public or academic environments. Additionally, younger orthodontists reported greater patient loads and higher income recovery.

Conclusions: The COVID-19 pandemic had a major impact on orthodontic practices in Europe, revealing regional differences in resilience and recovery. Key elements for ensuring continuity of care included infection control, digital transformation, and financial planning. These findings emphasize the necessity of developing localized support strategies and coordinated international responses to strengthen orthodontic practices in the event of future public health emergencies.

## Introduction

Background

The COVID-19 pandemic, caused by the SARS-CoV-2 virus, emerged in late 2019 and quickly transformed into a global health crisis, severely impacting healthcare systems worldwide. This situation resulted in significant disruptions across various healthcare sectors, particularly in dentistry, due to the increased risk of viral transmission associated with dental treatments. Procedures that generated aerosols presented substantial challenges, necessitating strict infection control measures and significant operational adjustments. Across the globe, dental practices faced enforced closures, restrictions on emergency services, modified infection control protocols, and significant financial impacts [1-3].

Orthodontists worldwide faced significant repercussions and encountered substantial operational challenges, necessitating adjustments to their practices. In the U.S., orthodontists experienced major disruptions, including a sharp decline in patient numbers, increased reliance on teleorthodontics, and financial difficulties that resulted in average income losses of approximately 50% [4,5]. Similarly, in India, orthodontists dealt with widespread clinic closures, a notable decrease in patient compliance, economic pressures, and a marked shift towards virtual consultations to ensure patient care [6,7]. In the Middle East, orthodontists faced significant operational challenges, adversely impacting their income and psychosocial well-being, contributing to issues such as anxiety and depression. Nevertheless, many respondents remained optimistic about the future of their profession and reported improvements in their social lives with family and friends due to increased free time [8]. In South America, particularly in Brazil, orthodontic practices encountered intensified financial challenges and increasingly relied on digital communication tools for patient management. Additionally, most patient appointments focused on emergency care, and the pandemic significantly impacted dental appointments and increased patient anxiety levels, demonstrating a clear connection between patients' emotional states and their willingness to attend dental visits [9,10].

The data presented above reveal the significant common challenges faced by the orthodontic profession worldwide. However, the varied regional impacts, approaches to response, and recovery pathways highlight the need for tailored assessments across different geographic areas to gain a deeper understanding of the unique experiences and adaptive methods.

Objectives

The present initiative, led by the European Federation of Orthodontics (FEO), aimed to explore, in a descriptive and exploratory manner, the impact of the COVID-19 pandemic on orthodontic practices in Europe. Specifically, the study evaluated four main domains: (1) clinical and operational adaptations, including changes in scheduling, infection-control procedures, ventilation, and treatment delivery; (2) financial consequences for practices; (3) patient-management and digital communication strategies, including teleorthodontic use; and (4) psychological and global perceptions, including stress, preparedness, perceived occupational risk, and anticipated long-term effects on orthodontic practice. The study was designed to describe trends and explore associations according to gender, country of practice, years in practice, practice type, and vaccination status.

## Materials and methods

Study design, setting, and participants

The information presented in this paper originates from a cross-sectional survey conducted among orthodontists who are members of national societies affiliated with the European Federation of Orthodontics from June to September 2022. Ethical approval was not required, as determined by the FEO COVID Committee, and participants were asked to provide informed consent for data collection and processing at the beginning of the survey (Table 5 [Appendix]). The questionnaire was reviewed by members of the FEO COVID Committee and representatives from participating national societies to ensure clarity, relevance, and consistency with previous international surveys. As the questions were factual and experience-based, a formal pilot test was not deemed necessary.

The questionnaire link was distributed to national orthodontic societies affiliated with the FEO, which then shared it among their members. Because of this decentralized dissemination, the total number of recipients could not be determined, and a precise response rate could not be calculated. The study, therefore, reflects a convenience sample of orthodontists who voluntarily responded. All data were collected and processed anonymously. Participants received a self-administered questionnaire via email. The survey, hosted on Google Forms (a GDPR-compliant platform), took approximately five minutes to complete and was structured into two sections.

In the first section, orthodontists provided demographic details and then answered questions regarding their experiences during the COVID-19 pandemic, including infection control strategies, pandemic-related impacts on practice and finances, patient management practices during this time, and broader questions (Table 5 [Appendix]). There was no time restriction for completion.

Variables, data sources, and statistical analysis

The collected data were summarized through calculations of central tendency, variability indices, and frequencies. After recoding ordinal variables, we assessed the impact of gender on respondents’ experiences related to the COVID-19 pandemic, focusing on practice and financial implications, management strategies, and answers to global questions, utilizing the Mann-Whitney test. We also examined the impact of country of practice, years in practice, practice type, and vaccination status using the Kruskal-Wallis test.

Given the exploratory nature of the study and the unequal group sizes across countries, only univariate non-parametric tests were performed. No correction for multiple comparisons or adjustment for confounding variables was applied, as the analyses were intended to describe associations. Consequently, the results should be interpreted as descriptive and indicative rather than inferential.

Respondents from countries with fewer than five responses were excluded from further analysis. The associations between nominal variable responses and factors such as gender, country of practice, years in practice, practice type, and vaccination status were analyzed using the chi-squared test. All statistical analyses were performed with IBM Corp. Released 2023. IBM SPSS Statistics for Windows, Version 29. Armonk, NY: IBM Corp. enhanced with the Exact Tests module for the Monte Carlo simulation method [11]. A significance level of α = 0.05 (p ≤ 0.05) was established for all hypotheses and testing procedures.

## Results

Participants and descriptive data

Of the total 436 responses, eight were excluded from further analysis because they came from countries with fewer than five respondents. From the remaining 428 orthodontists who completed the questionnaire, 168 were male (39.3%), and 260 were female (60.7%). Most respondents practiced in the United Kingdom (29.7%), Italy (28.2%), and Spain (19.2%), countries severely affected during the initial period of the COVID-19 pandemic (Table 1). More than half of the respondents (51.7%) are over 40 years old and have been practicing for over 20 years, with many currently serving as owners of orthodontic practices (52.2%). About 60% of respondents work for more than seven half-day treatment sessions, and 70% treat 10-20 patients per session (Table 2).

**Table 1:** Geographic distribution of respondents’ primary practice locations (N of responses [%]). *Countries with fewer than five responses were excluded from further analysis. The data have been represented as N, %. Statistical significance was not calculated in this table.

Country	# of responses (%)*
United Kingdom	127 (29.7)
Italy	121 (28.3)
Spain	82 (19.2)
Greece	46 (10.7)
Belgium	31 (7.3)
Ireland	12 (2.8)
Egypt	9 (2.1)
France	3
Luxembourg	1
North Macedonia	1
In Europe - association not in FEO	2
Outside Europe - association not in FEO	1

**Table 2:** Demographics of survey participants, including age, years in specialized practice, practice type, weekly treatment sessions (half-day), and patients treated per session (N of responses [%]).

Age		Years in practice		Type of practice		Sessions/week		Patients/session	
<30	8 (1.9)	<5	28 (6.6)	Practice owner	223 (52.2)	1-2	13 (3.0)	<10	126 (29.5)
30-39	70 (16.4)	6-10	52 (12.2)	Public sector	53 (12.4)	3-4	60 (14.1)	10-20	302 (70.5)
40-49	113 (26.5)	11-15	54 (12.6)	University clinic	16 (3.7)	5-6	101 (23.7)	>20	0 (0)
50-59	145 (33.9)	16-20	72 (16.9)	Practice associate	136 (31.7)	7-8	125 (29.3)		
>60	92 (21.5)	>20	222 (51.7)			9-10	129 (29.9)		

The vast majority of orthodontists who participated in the survey had completed their vaccinations, and all or most of their staff were fully vaccinated (Table 3). Two hundred forty-nine respondents (58.1%) had tested positive for COVID-19; the vast majority of them had done so once (87%) or twice (12%), with a minimal minority testing positive more than twice (1.0%). Testing positive led to staying out of practice for two weeks or less (91.2%), three to four weeks (7.6%), one to three months (0.4%), or more than three months (0.8%).

**Table 3:** Vaccination status among participating orthodontists and their staff (N of responses [%]).

Vaccination status -Orthodontists		Complete vaccination -Staff	
Vaccinated	79 (18.5%)	All of them	341 (79.6%)
Booster dose 1	107 (25.0%)	Most of them	75 (17.5%)
Booster dose 2	229 (53.5%)	50%	2 (0.4%)
Not vaccinated	13 (3.0%)	A few of them	2 (0.4%)
		None of them	8 (2.1%)

Outcome data and main results

Table 4 shows the effects of gender, country, years in practice, type of practice, and vaccination status on respondents’ experiences of working during the COVID-19 pandemic, including practice and financial implications, patient management strategies, and responses to global questions. While no statistically significant differences were noted based on gender or vaccination status, the country of practice appeared to have a statistically significant influence on the responses to the survey. Patients’ waiting lists increased more in the United Kingdom, while in Egypt, Spain, and Italy, they remained the same (Table 4 and Table 6 [Appendix]). Orthodontists from Greece reported feeling significantly more stressed during treatment delivery (mean=2.93±0.88), followed by those from the United Kingdom, Italy, Belgium, and Spain. In contrast, orthodontists from Egypt and Ireland reported the least stress (2.00±1.21). Staff in Greek orthodontic practices also felt significantly more stressed during treatment delivery (3.04±0.84), followed by those from Italy, the United Kingdom, Spain, Ireland, Belgium, and Egypt (2.22±0.97) (Table 4, and Tables 7, 8 [Appendix]).

**Table 4:** The effects of gender (Mann-Whitney), country, years of practice, type of practice, and vaccination status (Kruskal-Wallis) on respondents’ experiences during the COVID-19 pandemic, practice and financial implications, patient management strategies, and responses to global questions. Statistically significant differences are highlighted in bold (p<0.05). §Mann-Whitney test [Z, p-value]: gender; Kruskal-Wallis test [H, p-value]: country of primary practice, years in practice, type of practice, and vaccination status. §§X^2^ test [X^2^, p-value]

	Gender	Country of practice	Years in practice	Type of practice	Vaccination status
Experiences of working during the period of the COVID-19 pandemic					
What is your current patient load compared to the period before?^§^	0.207	0.071	<0.001	0.005	0.768
Has your waiting list increased compared to the period before?^§§^	0.527	<0.001	0.104	<0.001	0.653
Do you perceive treatment delivery to be more or less stressful compared to pre-pandemic?^§^	0.310	0.032	0.522	0.310	0.105
Does your staff perceive treatment delivery to be more or less stressful compared to pre-pandemic?^§^	0.836	0.033	0.715	0.426	0.596
Practice and financial implications of the COVID-19 pandemic					
How does your revenue from practicing orthodontics compare to before the COVID-19 pandemic?^§^	0.281	0.565	0.009	0.093	0.793
Have the COVID-19 related expenses significantly affected your practice?^§^	0.596	0.106	0.426	0.260	0.846
Has your practice financial status reverted to the pre-COVID-19 situation?^§^	0.132	0.867	0.111	0.553	0.594
Patient management strategies during the COVID-19 pandemic					
Was the progress of your patients’ treatment delayed as a result of the COVID-19 pandemic?^§§^	0.509	<0.001	0.508	<0.001	0.670
Have you compromised your standards of ‘finished result’ in the light of the COVID-19 pandemic?^§§^	0.901	0.002	0.981	0.001	0.309
Global Questions					
Do you consider yourself prepared for practicing Orthodontics until the COVID-19 pandemic ends?^§^	0.591	<0.001	0.383	0.104	0.174
Do you feel that you are at risk of COVID-19 infection because of practicing Orthodontics?^§^	0.960	0.003	0.244	0.621	0.471
Do you feel that the COVID-19 pandemic will permanently affect the practice of Orthodontics?^§^	0.521	0.035	0.317	0.256	0.098

In the United Kingdom, the treatment progress for patients was significantly delayed due to the COVID-19 pandemic, whereas in Greece and Italy, it remained essentially unchanged (Table 4 and Table 9 [Appendix]). Orthodontists in Greece, Ireland, and Belgium did not indicate any compromises in the quality of the ‘finished result'. In contrast, those from Egypt cited minor compromises, whereas orthodontists from Spain, the United Kingdom, and Italy reported substantial compromises (Table 4 and Table 10 [Appendix]).

Orthodontists in Greece rated their preparedness to practice during the ongoing COVID-19 pandemic higher (3.67±0.47) than their counterparts from Spain, the United Kingdom, Ireland, Italy, Egypt, and Belgium (3.00±0.82). Egyptian orthodontists felt a greater risk of COVID-19 infection associated with their practice (3.33±0.71) compared to those in Greece, Belgium, the United Kingdom, Italy, Spain, and Ireland (2.08±0.90). Lastly, Belgian doctors believed the COVID-19 pandemic would have a lasting impact on orthodontics (2.65±0.98), unlike their peers from Ireland, the United Kingdom, Greece, Spain, Italy, and Egypt (2.11±0.78) (Tables 7, 8 [Appendix]).

Furthermore, years of experience in orthodontics appeared to influence responses to some survey questions. The current patient load for those practicing orthodontics for 6 to 10 years (2.32±1.00) was higher compared to those practicing for over 20 years (1.65±0.92). Likewise, the revenue was greater in the former group (2.14±0.84) than in the latter (1.64±0.97) (Table 4 and Table 11 [Appendix]).

Regarding the type of practice, the patient load increased more among those practicing privately (Table 4 and Table 11 [Appendix]). In contrast, the waiting list grew more in the public sector (Table 4 and Table 12 [Appendix]), and the progress of patient treatment was delayed (Table 4 and Table 13 [Appendix]). Most participants in private practices upheld their standards for the 'finished result,' while many in hospital settings, the public sector, or university clinics made minor compromises (Table 4 and Table 14 [Appendix]).

**Figure 1:**
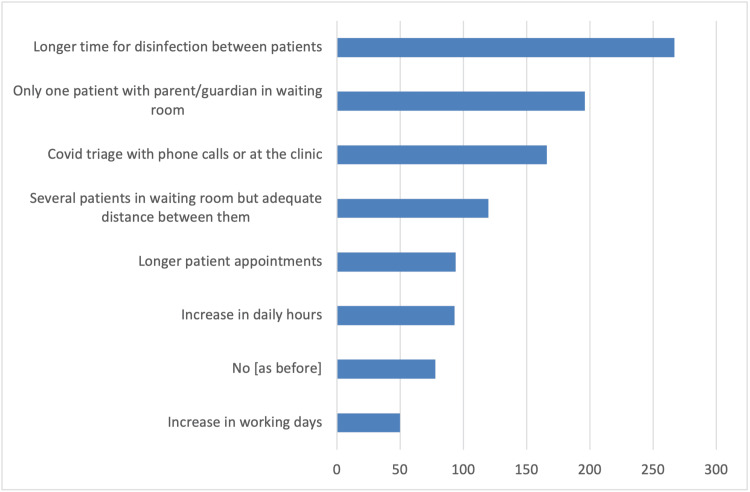
Responses to the question “Has the schedule/function of your clinic changed compared to the period before the COVID-19 pandemic?”. Several answers could be selected.

**Figure 2:**
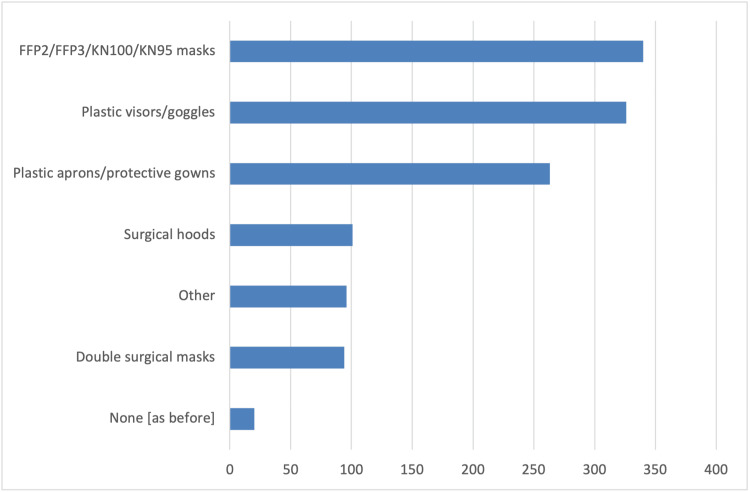
Responses to the question “What EXTRA personal protective equipment measures have you been taking because of the COVID-19 pandemic?”. Several answers could be selected.

**Figure 3:**
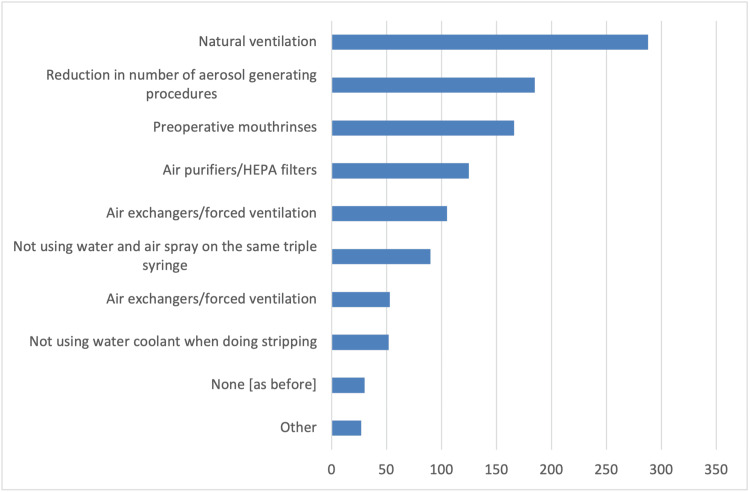
Responses to the question “What EXTRA measures have you been taking to increase air quality in your practice because of the COVID-19 pandemic?”. Several answers could be selected.

**Figure 4:**
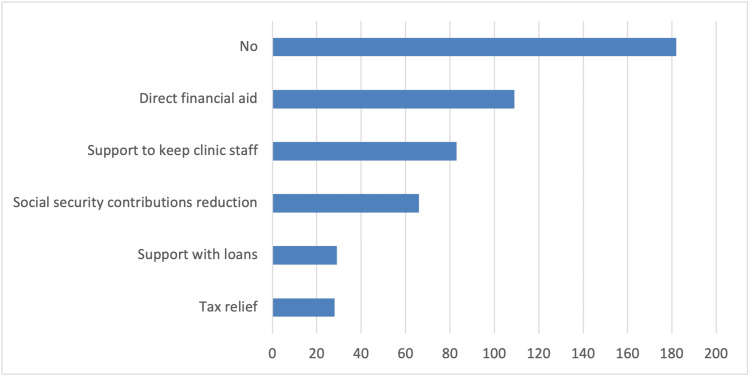
Responses to the question “Has your government provided economic aid or other form of support to orthodontists?”. Several answers could be selected.

**Figure 5:**
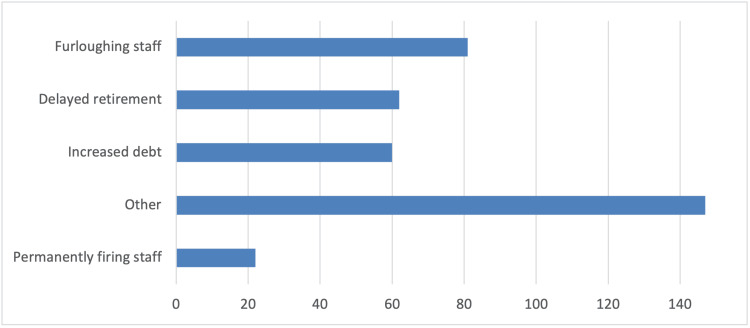
Responses to the question “Which of the following did you experience as negative consequences of the COVID-19 pandemic for your practice?”. Several answers could be selected.

**Figure 6:**
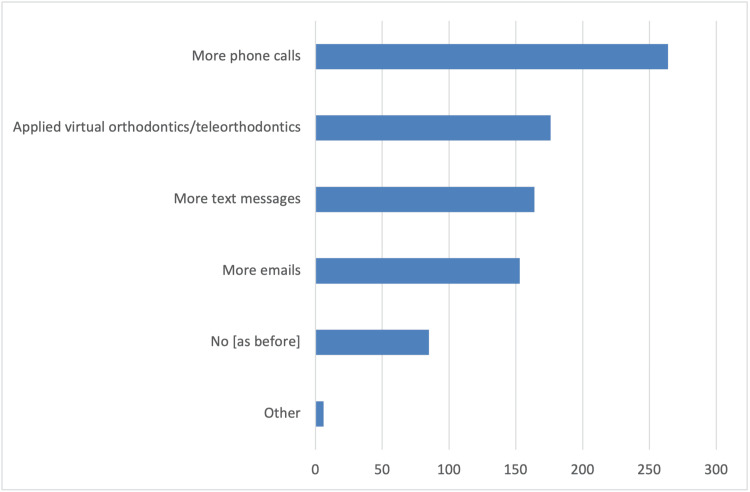
Responses to the question “Did the ways of contacting patients during the COVID-19 pandemic change?”. Several answers could be selected.

**Figure 7:**
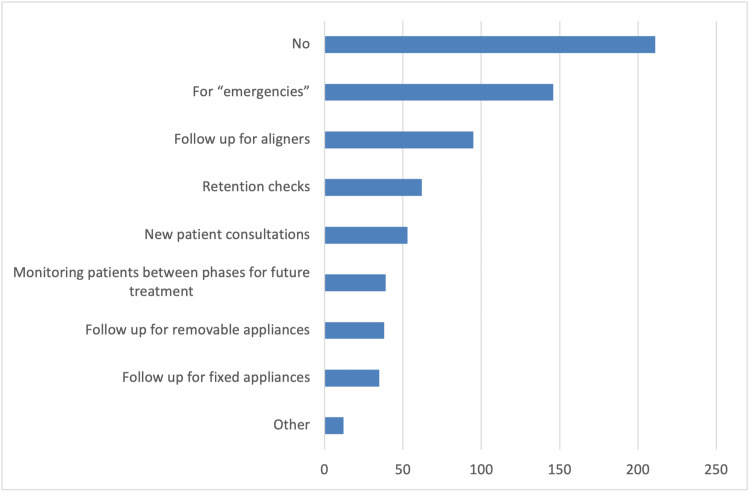
Responses to the question “Do you practice virtual orthodontics/teleorthodontics currently, and in which cases?”. Several answers could be selected.

In addition, survey participants reported several changes in the schedule and operation of their clinics compared to pre-COVID-19 pandemic times. These changes include longer patient appointments, allowing only one patient accompanied by a parent or guardian in the waiting room, and triage conducted via phone calls or at the clinic (Figure 1). As strategies to reduce infections, the vast majority of orthodontists in the survey reported using special masks, plastic visors or goggles, and plastic aprons or protective gowns as additional personal protective equipment measures (Figure 2). Furthermore, they primarily utilized natural ventilation to enhance air quality (Figure 3). Nearly half of the responding orthodontists did not receive any economic aid or support from the government to alleviate the practice and financial impacts of the COVID-19 pandemic (Figure 4). Negative consequences for practices included furloughing staff, delaying retirement, accumulating debt, and permanently terminating employees (Figure 5). Finally, regarding patient management strategies, most orthodontists reported modifications in how they communicated with patients, including an increased number of phone calls, text messages, and emails, as well as the use of teleorthodontics (Figure 6). The latter continues to be utilized for various purposes by many survey respondents (Figure 7).

## Discussion

Key results

The survey conducted by the European Federation of Orthodontics revealed significant effects of the COVID-19 pandemic on orthodontic practices throughout Europe. A total of 428 orthodontists participated, reporting considerable changes in clinical operations, financial conditions, and patient management. Practices implemented infection control measures and strategies to improve air quality, along with procedural adjustments. Financially, the lack of government assistance resulted in significant negative consequences. Modifications in communication methods became essential for managing emergencies, following up with patients, and carrying out consultations. Furthermore, treatment delays and variations in treatment quality were observed differently across countries. Stress levels among practitioners and staff showed marked differences. This survey highlights the major changes and challenges faced by European orthodontists during the COVID-19 pandemic, emphasizing the need for ongoing support and strategic planning to effectively tackle similar challenges in the future.

The COVID-19 pandemic brought significant changes to the professional environment for orthodontists worldwide, and the European Federation of Orthodontics (FEO) survey revealed considerable geographic variations. Orthodontists in Europe faced reduced patient volumes, increased stress, longer appointment times, and substantial alterations to clinical workflows. In the United Kingdom, significant increases in patient waiting lists and appointment delays were reported, while orthodontic practice in Greece and Ireland experienced fewer similar effects. These results align with various international studies, although the degree of impact varied due to geographical, infrastructural, and governmental factors. For example, a study from France [12] indicated that although infection control measures became routine, patient flow did not change significantly, and schedule organization adapted accordingly. In Poland, research by Sycińska-Dziarnowska et al. (2022) indicated that over 85% of orthodontists had to temporarily close their practices and were only accepting patients with emergencies [13]. In Finland, Riekkinen et al. (2023) noted that preventive measures and adjustments to treatment protocols were implemented in response to the local COVID-19 situation [14].

Delays in treatment were a widespread concern due to lockdowns or patients' worries about contracting COVID-19 during their treatment visits. FEO respondents from the UK experienced longer treatment times compared to their counterparts in Italy. Similar findings were reported in countries outside of Europe. During the COVID-19 pandemic in Jordan, patients undergoing active orthodontic treatment were prioritized, often at the expense of delaying care for new and follow-up patients [15]. Additionally, treatment times were also found to be longer in South India [7,16]. The extended duration of treatment may have also influenced the objectives set by orthodontists and patients. In this context, the European Federation of Orthodontics (FEO) survey indicated that a significant number of orthodontists, particularly in the UK, reported having compromised their treatment standards due to challenges associated with the pandemic. In contrast, orthodontists in countries like Greece, Ireland, and Italy reported fewer compromises, potentially attributed to less stringent lockdown measures or better continuity of care. With the decline in face-to-face consultations, alternative methods for patient interaction were introduced. During the pandemic, orthodontists significantly increased their use of phone calls, texts, and emails, as well as teleorthodontic services, to stay connected with patients. Nevertheless, the use of this strategy decreased after the peak of the pandemic. Many respondents noted that by the time they completed the FEO survey, they had stopped using teleorthodontics, with only a few still utilizing it for follow-ups on fixed appliances.

In countries outside of Europe, similar operational disruptions and changes to those described above were observed. Motevasel et al. (2021) emphasized the adverse effects of mandatory office closures in the USA, with treatment delays being the most frequently reported consequence [4]. In India, both Kumar and Sharma (2021) [6] and George et al. (2021) [17] reported that many orthodontists drastically reduced their clinical activities, with many opting for teledentistry during lockdowns. Additionally, Dhanasekaran et al. (2021) found that elective procedures had been deferred, and patients were managed through virtual teleconsultations, a phenomenon also seen in Europe, as mentioned above [18].

Regarding stress and psychological strain, the FEO survey revealed that orthodontists in Greece and the UK experienced particularly high levels of stress. Similar observations were made in Turkey, where Yilmaz and Ozbilen (2020) reported instances of anxiety, especially concerning individuals who perceived those around them as lacking sufficient knowledge about COVID-19 and failing to adhere to hygiene guidelines [19]. Another study conducted in Iraq and Turkey indicated that the situation was similar among Turkish and Iraqi orthodontists, whose psychological status was likely affected by concerns about the virus posing a threat to their health and the well-being of their families. In Jordan, Sabbagh et al. (2023) recorded that stress during the pandemic, especially during lockdowns, contributed to increased psychological pressure on patients, leading to suboptimal compliance with orthodontic instructions [15]. A study from Pakistan (Shaukat et al., 2023) echoed these findings, reporting that most practitioners experienced a negative impact on their psychological well-being, with a statistically significant difference in the responses of male and female professionals [20]. In contrast to these findings, Irish and Egyptian orthodontists in the FEO survey reported lower levels of stress.

Moreover, the COVID-19 pandemic significantly impacted infection control protocols in orthodontic practices. According to a survey conducted by the European Federation of Orthodontics (FEO), practices across Europe rapidly adopted enhanced protective measures. A primary strategy emphasized in the FEO survey was the increased use of personal protective equipment (PPE), including FFP2/FFP3 masks, protective visors, and disposable gowns. The adoption of these PPE protocols grew considerably during the pandemic and continued afterward in many instances. This aligns with findings from France, where Loiseau et al. (2024) noted that most orthodontists continued to wear FFP2 masks even post-pandemic [12]. Similar trends were observed in Poland, where more than 90% of practitioners implemented and maintained strict PPE protocols during the peak of the pandemic [13]. Finland also reported high compliance rates with PPE usage, including face masks, gloves, disposable head covers, and additional protective clothing [14]. Lamb et al. (2023) suggested that although the extensive use of enhanced PPE might decline post-pandemic, practices such as high-volume suction are likely to remain standard during aerosol-generating procedures. In contrast, the use of teleorthodontics is anticipated to remain limited [21].

Natural ventilation was the most common environmental control method among the surveyed individuals. According to Loiseau and coworkers, besides improving ventilation, the organization of practices remained unchanged even after the pandemic peaked. This included using protective screens, filtration systems, hand sanitizers, and designated travel paths, and removing magazines [12]. Additionally, the FEO survey observed a significant decrease in aerosol-generating procedures. Similarly, in Finland, orthodontists limited the use of turbines, ultrasonics, water-air spray, and micromotors [14]. The importance of reducing aerosol production was emphasized again in a recent systematic review as well [22].

Another relevant finding identified by the FEO survey was that orthodontists shared a common belief that orthodontic practice has undergone permanent changes due to the pandemic, particularly in the UK and Belgium. Similar opinions were expressed in Turkey, where Yilmaz and Ozbilen (2020) found that the majority of participants would continue using additional PPE, such as N95 masks and face shields, as part of their routine practice after the outbreak, although at a lower rate compared to usage during the COVID-19 pandemic [19]. Furthermore, practitioners both in the United Kingdom and Jordan acknowledged that the pandemic would have a lasting impact on both clinical services and society [15,23].

The COVID-19 pandemic placed unprecedented economic pressure on orthodontic practices worldwide. The FEO survey indicated that most orthodontists experienced a decline in income and increased expenses during this period. This aligns with findings from the United States, where Motevasel et al. (2021) noted income reductions of about 50% across all demographic categories [4]. A similar decrease in total income was also noted in a study conducted in South India [16]. In Brazil, Cotrin et al. (2020) reported significant concerns regarding the financial impact among the majority of survey participants [9]. Additionally, nearly half of the respondents in the FEO survey mentioned that they did not receive financial support from their governments [9]. This lack of support resulted in greater debt, delayed retirement plans, employee furloughs, and, in some cases, permanent layoffs. Meanwhile, in the United States, approximately 93% of practices applied for and received some form of stimulus funding provided by the Coronavirus Aid, Relief, and Economic Security (CARES) Act [4].

The European Federation of Orthodontics (FEO) survey also explored broader global reflections on the COVID-19 pandemic, focusing on perceived preparedness, perceived risk, and anticipated long-term effects on orthodontic practices. Orthodontists in Greece rated their preparedness to handle the current pandemic higher than their counterparts in other countries, while Egyptian orthodontists perceived a greater risk of COVID-19 infection linked to their work.

The impact of COVID-19 on orthodontic practice varied not only by country but also based on the experience of practicing orthodontics. The FEO survey revealed that younger orthodontists, especially those with 6 to 10 years of experience, handled a higher patient load and generated more revenue than those practicing for over 20 years at the time of the survey. This could suggest that younger orthodontists may be more adaptable and possibly more skilled in using digital and teleorthodontic tools. A study in Italy observed that orthodontic emergencies were relatively rare, with many managed via telephone consultation. The incidence of emergencies caused by removable appliances, such as clear aligners, was much lower compared to fixed appliances like multibracket systems. This difference likely contributed to most clinicians choosing removable appliances during the COVID-19 pandemic [24]. Research by Sycińska-Dziarnowska et al. (2022) [13] and George et al. (2021) [17] showed that orthodontic practitioners were familiar with teledentistry, with younger practitioners showing a more positive attitude toward its implementation. Additionally, a systematic review concluded that teleorthodontics can be a valuable tool for managing orthodontic emergencies [25].

Another key factor was the type of practice involved. Those in private practice observed a greater increase in patient numbers. On the other hand, the public sector experienced a notable rise in waiting lists, leading to delays in patient care. Most private practice participants upheld their standards for the 'finished result,' while numerous professionals in hospitals, public settings, or university clinics made minor compromises. These results may be linked to the greater flexibility in modifying workflows and patient management strategies seen in private practices. Conversely, practitioners in the public sector and academia might have faced more bureaucratic obstacles when resuming clinical activities. Finally, respondents' vaccination status did not affect their experiences working during the COVID-19 pandemic, which included practical and financial consequences as well as patient management strategies. In Jordan, most respondents supported the vaccination of orthodontic staff and were optimistic about the impact of a vaccination program on restoring clinical services [15].

The present survey presents a cautious yet informative analysis of how the COVID-19 pandemic has impacted orthodontic practices across Europe. The results reveal significant clinical, operational, and financial disruptions, which varied considerably between countries, practice types, and practitioner experience levels. While these findings should be considered in light of the limitations mentioned earlier, they are consistent with evidence from similar studies worldwide. This alignment supports the validity of the information provided and highlights the role of local context in shaping experiences and recovery under similar circumstances.

Clinical significance

This study’s findings suggest several proactive strategies to effectively prepare the orthodontic community for comparable public health emergencies in the future. Firstly, there is a need for comprehensive emergency preparedness protocols that include clinical guidelines, standardized personal protective equipment usage, and training in infection control. Secondly, the acceleration of digital transformation is crucial. The adoption of teleorthodontics during the pandemic highlights the necessity for increased investment in virtual care platforms, data security, and remote monitoring tools. Thirdly, boosting economic resilience is vital, which can be achieved by diversifying income streams and providing financial preparedness training. Lastly, fostering international collaboration, data sharing, and joint research initiatives can help quickly adapt best practices across different regions. A unified professional front will be critical in efficiently addressing global health threats with agility and coherence.

Limitations of the study

Although this study provides significant insights into the impact of the COVID-19 pandemic on orthodontic practices throughout Europe, it is essential to recognize some limitations. First, the research employed a convenience sampling method, potentially restricting the generalizability of its findings. The use of a voluntary convenience sample limits the representativeness of the study population and reduces the full reproducibility of the sampling approach. Respondents may have held stronger opinions or encountered more pronounced experiences regarding the pandemic, which could introduce selection bias. Second, while the survey collected responses from 428 orthodontists across Europe, the distribution was uneven across countries, particularly skewed towards the United Kingdom, Italy, and Spain. This geographic disparity may distort regional comparisons and underrepresent the experiences of practitioners in other regions. Moreover, since the responses were self-reported, there is a risk of recall bias or social desirability bias, especially concerning sensitive issues like financial difficulties or compromises in patient care [26]. In addition, the survey instrument was not formally validated; however, it was developed and reviewed by the FEO COVID Committee and national society representatives based on existing international questionnaires to ensure content validity. We do acknowledge that the self-reported and retrospective nature of the responses may have introduced recall or selection bias. Also, the statistical analysis was limited to univariate, non-parametric comparisons without adjustment for multiple testing or control of confounders. While this approach was appropriate for the exploratory scope of the study, it limits the strength of inferences that can be drawn. Furthermore, the lack of a calculable response rate and the unequal national representation limit the generalizability of cross-country comparisons. The results should therefore be interpreted as descriptive observations rather than inferential comparisons. Apart from that, the study’s cross-sectional design restricts the ability to establish causal relationships or monitor long-term outcomes. Finally, the lack of a control or comparator group (e.g., general dentists or healthcare workers from other specialties) limits the context of these findings within the broader healthcare landscape. In addition, although the questionnaire was developed with expert input and based on prior international surveys, it was not formally validated, and no pilot testing was performed. Despite these limitations, the study offers a valuable snapshot of the pandemic-related challenges faced by European orthodontists and identifies areas needing ongoing support and strategic planning for better future responses to similar challenges.

## Conclusions

During the COVID-19 pandemic, European orthodontists reported challenges similar to those described in other regions, including reduced clinical activity, changes in patient management, financial strain, and increased stress. Within this survey, respondents also described variations in recovery, preparedness, and perceived impact across countries and practice settings. However, given the exploratory cross-sectional design, convenience sampling, and descriptive analytical approach, these findings should be interpreted as indicative perceptions and observed trends, rather than definitive evidence of regional differences or long-term systemic effects.

The survey findings suggest that the pandemic was associated with substantial operational and financial disruption, including reported income reduction, limited financial support in many settings, and uneven recovery patterns according to geography, practice type, and professional context. The results also indicate that the pandemic accelerated the use of digital communication and teleorthodontic approaches in patient management. Although remote strategies appeared useful for maintaining communication and supporting selected aspects of care, respondents also reported limitations in their clinical applicability. Accordingly, teleorthodontics may remain a valuable adjunct to practice but not a replacement for comprehensive in-person orthodontic treatment. Overall, the findings point to the potential importance of preparedness planning, adaptable infection-control procedures, financial resilience, and context-sensitive support strategies for future public health emergencies. At the same time, because this study was descriptive and exploratory, the observed geographic and professional differences should be interpreted cautiously and confirmed in future research using more robust sampling and analytical designs.

## Appendices

Impact of the COVID-19 pandemic on the professional practice of orthodontists in Europe

This survey aims to explore the effects and consequences of the COVID-19 pandemic on the professional practice of orthodontists in Europe and is intended for clinically active orthodontists that have been practicing during the period of the COVID-19 pandemic.

Your participation in the survey is important. Only anonymized data will be collected and will be processed as a whole. If you have any questions, please contact xxxxxx@feo-online.com

If you have been practicing orthodontics during the period of the COVID-19 pandemic and consent to participate in this survey, choose “Yes”. Otherwise, choose “No”.

Yes; No

**Table 5:** Questionnaire regarding the Impact of the COVID-19 pandemic on the professional practice of orthodontists in Europe

Section 1: Demographic information	
Which country do you work in?	Albania; Belgium; Bulgaria; Croatia; Cyprus; France; Germany; Greece; Ireland; Italy; Luxembourg; North Macedonia; Portugal; Romania; Serbia; Slovenia; Spain; Turkey; United Kingdom; Egypt; Israel; Lebanon
What is your gender?	Male; female; other
What is your age?	<30; 30-39 years; 40-49 years; 50-59 years; >60 years
How many years have you been practicing as a specialist orthodontist?	<5 years; 6-10 years; 11-15 years; 16-20 years; >20 years
Where do you primarily work?	Orthodontic practice (owner); Orthodontic practice (associate); Hospital setting/Public sector; University clinic
How many patient treatment sessions (half day) do you have on average per week?	1-2; 3-4; 5-6; 7-8; 9-10
How many patients do you treat per treatment session (half day)?	<10; 10-20; >20
What is your COVID-19 vaccination status?	Not vaccinated; Vaccinated; Booster dose 1; Booster dose 2
Have you tested positive for COVID-19?	Yes; No
For how long did you stay out of your practice because of testing positive for COVID-19?	2 weeks or less; 3 to 4 weeks; 1 to 3 months; more than 3 months
Are your staff fully vaccinated against COVID-19?	None of them; a few of them; 50%; most of them; all of them
Section 2: Experiences of working during the period of the COVID-19 pandemic	
What is your current patient load compared to the period before the COVID-19 pandemic?	Far fewer; slightly reduced; as before; slightly more; many more
Has your waiting list increased compared to the period before the COVID-19 pandemic?	No; <3 months increase; 4-6 months; >7 months increase; not applicable
Do you perceive treatment delivery to be more or less stressful compared to pre- pandemic?	Much less stressful; slightly less stressful; as before; slightly more stressful; much more stressful
Does your staff perceive treatment delivery to be more or less stressful compared to pre- pandemic?	Much less stressful; slightly less stressful; as before; slightly more stressful; much more stressful
Has the schedule/function of your clinic changed compared to the period before the COVID-19 pandemic? [choose as many as they apply]	No [as before]; increase in daily hours; increase in working days; longer patient appointments; longer time for disinfection between patients; only one patient with parent/guardian in waiting room; several patients in waiting room but adequate distance; COVID triage with phone calls or at the clinic; other [please specify]
Section 3: Strategies to reduce infection	
What EXTRA personal protective equipment measures have you been taking because of the COVID-19 pandemic? [choose as many as they apply]	None [as before]; plastic aprons/protective gowns; double surgical masks; FFP2/FFP3/KN100/KN95 masks; surgical hoods with their own cooling fan; plastic visors/goggles; other [please specify at the comments section at the end of the survey]
What EXTRA measures have you been taking to increase air quality in your practice because of the COVID-19 pandemic? [choose as many as they apply]	None [as before]; natural ventilation; air exchangers/forced ventilation; air purifiers/HEPA filters; preoperative mouthrinses; reduction in number of aerosol generating procedures; not using water and air spray on the same triple syringe; not using water coolant when doing stripping; other [please specify at the comments section at the end of the survey]
Section 4: Practice and financial implications of the COVID-19 pandemic	
How does your revenue from practicing orthodontics compare to before the COVID-19 pandemic?	Much less; slightly less; as before; slightly more; much more; Not applicable
Has your government provided economic aid or other form of support to orthodontists? [choose as many as they apply]	No; Direct financial aid; Tax relief; Social security contributions reduction; Support to keep clinic staff; Support with loans; Not applicable
Have the COVID-19 related expenses significantly affected your practice?	Not at all affected; slightly affected; affected significantly; Not applicable
Which of the following did you experience as negative consequences of the COVID-19 pandemic for your practice? [choose as many as they apply]	Furloughing staff; permanently firing staff; increased debt; delayed retirement; none of the above; other [please specify]; Not applicable
Has your practice financial status reverted to the pre COVID-19 situation?	Not at all; slightly; somewhat; almost; completely; Not applicable
Section 5: Patient management strategies during the COVID-19 pandemic	
Did the ways of contacting patients during the COVID-19 pandemic change? [choose as many as they apply]	No [as before]; Applied virtual orthodontics/teleorthodontics; More emails; More phone calls; More text messages; Other [please specify at the comments section at the end of the survey]
Do you practice virtual orthodontics/teleorthodontics currently and in which cases? [choose as many as they apply]	No; New patient consultations; Follow up for aligners; Follow up for fixed appliances; Follow up for removable appliances; Monitoring patients between phases for future treatment; Retention checks; For “emergencies”; Other [please specify at the comments section at the end of the survey]
Was the progress of your patients’ treatment delayed as a result of the COVID-19 pandemic?	Not at all delayed; slightly delayed; significantly delayed
Have you compromised your standards of 'finished result' in the light of the COVID-19 pandemic?	Not at all compromised; slightly compromised; significantly compromised
Section 6: Global Questions & Comments	
Do you consider yourself prepared for practicing Orthodontics until the COVID-19 pandemic ends?	Not at all; slightly; somewhat; extremely
Do you feel that you are at risk of COVID-19 infection because of practicing Orthodontics?	Not at all; slightly; somewhat; extremely
Do you feel that the COVID-19 pandemic will permanently affect the practice of Orthodontics?	Not at all; slightly; somewhat; extremely

**Table 6:** Responses regarding the question, “Has your waiting list increased compared to the period before the COVID-19 pandemic?” are categorized by the country of primary practice (N of responses [% within the row], excluding “not applicable” responses).

Country	No	<3 months	4-6 months	>7 months	Total
Belgium	10 (35.7%)	7 (25.0%)	7 (25.0%)	4 (14.3%)	28 (100%)
Egypt	7 (87.5%)	0 (0.0%)	0 (0.0%)	1 (12.5%)	8 (100%)
Greece	24 (58.5%)	17 (41.5%)	0 (0.0%)	0 (0.0%)	41(100%)
Ireland	1 (8.3%)	6 (50.0%)	2 (16.7%)	3 (25.0%)	12 (100%)
Italy	83 (74.8%)	23 (20.7%)	4 (3.6%)	1 (0.9%)	111 (100%)
Spain	58 (74.4%)	19 (24.4%)	1 (1.3%)	0 (0.0%)	78 (100%)
United Kingdom	21 (17.1%)	19 (15.4%)	31 (25.2%)	52 (42.3%)	123 (100%)
Total	204 (50.9%)	91 (22.7%)	45 (11.2%)	61 (15.2%)	401 (100%)

**Table 7:** Respondents’ experiences while working during the COVID-19 pandemic, including practice and financial impacts, patient management strategies, and responses to global questions, were analyzed based on gender and country of practice (mean ± standard deviation).

	What is your current patient load compared to the period before the COVID-19 pandemic?	Do you perceive treatment delivery to be more or less stressful compared to pre- pandemic?	Does your staff perceive treatment delivery to be more or less stressful compared to pre- pandemic?	How does your revenue from practicing orthodontics compare to before the COVID-19 pandemic?	Have the COVID-19 related expenses significantly affected your practice?	Has your practice financial status reverted to the pre-COVID-19 situation?	Do you consider yourself prepared for practicing Orthodontics until the COVID-19 pandemic ends?	Do you feel that you are at risk of Covid-19 infection because of practicing Orthodontics?	Do you feel that the Covid-19 pandemic will permanently affect the practice of Orthodontics?
Gender									
Male	1.81±1.01	2.51±0.91	2.68±0.87	1.86±1.07	3.29±0.65	2.28±1.48	3.38±0.76	2.34±0.97	2.30±0.90
Female	1.90±0.95	2.60±0.91	2.67±0.84	1.75±0.92	3.34±0.60	2.03±1.46	3.37±0.70	2.35±1.00	2.25±0.92
Country									
Spain	2.06±0.95	2.44±0.83	2.61±0.73	1.87±0.88	3.20±0.55	2.02±1.54	3.54±0.76	2.13±1.07	2.12±1.00
United Kingdom	1.81±1.01	2.59±0.95	2.65±0.86	1.74±1.12	3.29±0.68	2.23±1.60	3.43±0.60	2.31±0.88	2.39±0.81
Italy	1.75±0.87	2.59±0.88	2.68±0.91	1.75±0.88	3.34±0.64	2.12±1.40	3.19±0.80	2.30±1.00	2.12±0.87
Egypt	1.33±1.00	2.00±1.22	2.22±0.97	1.13±0.99	3.50±0.55	1.60±1.82	3.11±0.78	3.33±0.71	2.11±0.78
Ireland	2.25±1.06	2.00±1.21	2.58±1.00	2.00±1.00	3.30±0.48	2.75±0.89	3.33±0.65	2.08±0.90	2.42±0.79
Greece	1.83±1.23	2.93±0.88	3.04±0.84	1.88±1.18	3.53±0.59	2.09±1.44	3.67±0.47	2.63±1.04	2.33±1.08
Belgium	1.97±0.75	2.58±0.76	2.52±0.72	1.80±0.76	3.22±0.64	2.23±1.51	3.00±0.82	2.61±0.88	2.65±0.98

**Table 8:** Experiences of respondents working during the COVID-19 pandemic, along with their responses to global questions, categorized by country of practice (pairwise comparisons; p<0.05). p-value: 0.05

Do you perceive treatment delivery to be more or less stressful compared to pre-pandemic?						
	United Kingdom	Italy	Egypt	Ireland	Greece	Belgium
Spain	0.296	0.348	0.207	0.188	0.003	0.670
United Kingdom		0.901	0.123	0.097	0.038	0.791
Italy			0.122	0.098	0.027	0.849
Egypt				0.972	0.026	0.188
Ireland					0.013	0.183
Greece						0.061
Does your staff perceive treatment delivery to be more or less stressful compared to pre- pandemic?						
	United Kingdom	Italy	Egypt	Ireland	Greece	Belgium
Spain	0.733	0.516	0.154	0.990	0.003	0.393
United Kingdom		0.742	0.157	0.883	0.007	0.324
Italy			0.145	0.782	0.019	0.253
Egypt				0.422	0.018	0.371
Ireland					0.139	0.718
Greece						0.004
Do you consider yourself prepared for practicing Orthodontics until the COVID-19 pandemic ends?						
	United Kingdom	Italy	Egypt	Ireland	Greece	Belgium
Spain	0.047	<0.001	0.054	0.156	0.625	<0.001
United Kingdom		0.026	0.194	0.598	0.020	0.005
Italy			0.688	0.649	<0.001	0.209
Egypt				0.554	0.022	0.775
Ireland					0.074	0.277
Greece						<0.001
Do you feel that you are at risk of COVID-19 infection because of practicing Orthodontics?						
	United Kingdom	Italy	Egypt	Ireland	Greece	Belgium
Spain	0.129	0.228	0.002	0.991	0.012	0.023
United Kingdom		0.930	0.001	0.353	0.043	0.087
Italy			0.003	0.449	0.058	0.119
Egypt				0.006	0.061	0.040
Ireland					0.084	0.096
Greece						0.811
Do you feel that the COVID-19 pandemic will permanently affect the practice of Orthodontics?						
	United Kingdom	Italy	Egypt	Ireland	Greece	Belgium
Spain	0.027	0.854	0.911	0.250	0.298	0.016
United Kingdom		0.012	0.350	0.732	0.690	0.173
Italy			0.954	0.190	0.275	0.008
Egypt				0.382	0.594	0.177
Ireland					0.755	0.565
Greece						0.206

**Table 9:** Responses to the question “Was your patients’ treatment delayed as a result of the COVID-19 pandemic?” distributed by the country of primary practice (N of responses [% within row]).

Country	Significantly delayed	Slightly delayed	Not at all delayed	Total
Belgium	6 (19.4%)	21 (67.7%)	4 (12.9%)	31 (100%)
Egypt	1 (11.1%)	6 (66.7%)	2 (22.2%)	9 (100%)
Greece	8 (17.4%)	25 (54.3%)	13 (28.3%)	46 (100%)
Ireland	3 (25.0%)	8 (66.7%)	1 (8.3%)	12 (100%)
Italy	10 (8.3%)	82 (67.8%)	29 (24.0%)	121 (100%)
Spain	9 (11.0%)	57 (69.5%)	16 (19.5%)	82 (100%)
United Kingdom	57 (44.9%)	68 (53.5%)	2 (1.6%)	127 (100%)
Total	94 (22.0%)	267 (62.4%)	67 (15.7%)	428 (100%)

**Table 10:** Distribution of responses to the question, “Have you compromised your standards of 'finished result' in the light of the COVID-19 pandemic?” based on the country of primary practice (N of responses [% within the row]).

Country	Significantly compromised	Slightly compromised	Not at all compromised	Total
Belgium	0 (0.0%)	6 (19.4%)	25 (80.6%)	31 (100%)
Egypt	0 (0.0%)	6 (66.7%)	3 (33.3%)	9 (100%)
Greece	0 (0.0%)	7 (15.2%)	39 (84.8%)	46 (100%)
Ireland	0 (0.0%)	2 (16.7%)	10 (83.3%)	12 (100%)
Italy	1 (0.8%)	28 (23.1%)	92 (76.0%)	121 (100%)
Spain	7 (8.5%)	20 (24.4%)	55 (67.1%)	82 (100%)
United Kingdom	3 (2.4%)	50 (39.4%)	74 (58.3%)	127 (100%)
Total	11 (2.6%)	119 (27.8%)	298 (69.6%)	428 (100%)

**Table 11:** Respondents’ experiences during the COVID-19 pandemic, including practice and financial implications, patient management strategies, and responses to global questions, according to years in practice, type of practice, and vaccination status (mean ± standard deviation).

	What is your current patient load compared to the period before the COVID-19 pandemic?	Do you perceive treatment delivery to be more or less stressful compared to pre- pandemic?	Does your staff perceive treatment delivery to be more or less stressful compared to pre- pandemic?	How does your revenue from practicing orthodontics compare to before the COVID-19 pandemic?	Have the COVID-19 related expenses significantly affected your practice?	Has your practice financial status reverted to the pre COVID-19 situation?	Do you consider yourself prepared for practicing Orthodontics until the COVID-19 pandemic ends?	Do you feel that you are at risk of COVID-19 infection because of practicing Orthodontics?	Do you feel that the COVID-19 pandemic will permanently affect the practice of Orthodontics?
Years in practice									
<5	2.10±1.01	2.34±0.81	2.55±0.74	1.82±1.24	3.29±0.69	2.21±1.76	3.34±0.67	2.66±0.90	2.00±0.80
6-10	2.32±1.00	2.70±0.75	2.72±0.72	2.14±0.84	3.14±0.59	2.26±1.55	3.45±0.67	2.17±0.85	2.30±0.95
11-15	2.05±1.02	2.57±0.87	2.66±0.84	2.07±0.96	3.40±0.55	2.56±1.42	3.39±0.73	2.38±1.02	2.45±0.89
16-20	1.96±0.92	2.60±1.00	2.59±0.88	1.82±0.97	3.33±0.63	2.36±1.53	3.51±0.60	2.26±0.97	2.32±0.90
>20	1.65±0.92	2.55±0.94	2.71±0.89	1.64±0.97	3.33±0.64	1.95±1.41	3.30±0.77	2.37±1.01	2.24±0.92
Type of practice									
Orthodontic practice (owner)	1.89±0.98	2.57±0.93	2.70±0.87	1.87±0.99	3.36±0.63	2.13±1.45	3.44±0.73	2.30±1.03	2.22±0.95
Hospital setting/Public sector	1.70±0.91	2.66±0.78	2.70±0.72	2.00±1.00	3.22±0.83	1.88±1.55	3.32±0.73	2.30±0.87	2.49±0.80
University clinic	1.22±0.65	2.11±1.13	2.33±1.03	1.00±0.89	3.00±0.63	1.40±1.52	3.33±0.69	2.44±0.92	2.17±0.92
Orthodontic practice (associate)	1.99±0.99	2.59±0.89	2.66±0.85	1.69±0.96	3.25±0.59	2.26±1.54	3.28±0.71	2.42±0.95	2.28±0.88
Vaccination status									
Vaccinated	1.95±0.98	2.66±0.89	2.72±0.84	1.71±1.00	3.36±0.57	2.26±1.49	3.48±0.69	2.30±1.04	2.21±0.91
Booster dose 1	1.79±1.05	2.59±0.96	2.72±0.88	1.78±1.05	3.33±0.65	2.16±1.42	3.28±0.71	2.38±0.96	2.34±0.9
Booster dose 2	1.87±0.92	2.56±0.86	2.64±0.84	1.84±0.96	3.28±0.64	2.12±1.50	3.37±0.74	2.37±0.97	2.29±0.92
Not vaccinated	1.92±1.31	1.83±1.27	2.50±1.09	1.82±0.75	3.40±0.52	1.60±1.35	3.50±0.80	1.92±1.16	1.67±0.78

**Table 12:** Responses regarding the question “Has your waiting list increased compared to the period before?” categorized by the participants’ practice type (N of responses [% within row], excluding “not applicable” responses).

Type of practice	No	<3 months	4-6 months	>7 months	Total
Orthodontic practice (owner)	118 (56.0%)	53 (25.0%)	14 (7.0%)	25 (12.0%)	210 (100.0%)
Hospital setting/Public sector	9 (18.0%)	8 (16.0%)	13 (25.0%)	21 (41.0%)	51 (100.0%)
University clinic	7 (46.0%)	4 (27.0%)	1 (7.0%)	3 (20.0%)	15 (100.0%)
Orthodontic practice (associate)	73 (55.0%)	30 (22.0%)	17 (13.0%)	13 (10.0%)	133 (100.0%)
Total	207 (50.0%)	95 (24.0%)	45 (11.0%)	62 (15.0%)	409 (100.0%)

**Table 13:** Distribution of responses to the question “Was the progress of your patients’ treatment delayed as a result of the COVID-19 pandemic?” categorized by participants’ primary job (N of responses [% within row]).

Type of practice	Significantly delayed	Slightly delayed	Not at all delayed	Total
Orthodontic practice (owner)	39 (17.5%)	137 (61.5%)	47 (21.0%)	223 (100.0%)
Hospital setting/ Public sector	32 (60.0%)	21 (40.0%)	0 (0.0%)	53 (100.0%)
University clinic	2 (12.5%)	9 (56.0%)	5 (31.5%)	16 (100.0%)
Orthodontic practice (associate)	22 (16.0%)	97 (71.5%)	17 (12.5%)	136 (100.0%)
Total	95 (22.0%)	264 (62.0%)	69 (16.0%)	428 (100.0%)

**Table 14:** Distribution of responses to the question “Have you compromised your standards of 'finished result' in the light of the COVID-19 pandemic?” based on participants’ practice type (N of responses [% within row]).

Type of practice	Significantly compromised	Slightly compromised	Not at all compromised	Total
Orthodontic practice (owner)	4 (2.0%)	48 (21.5%)	171 (76.5%)	223 (100.0%)
Hospital setting/Public sector	2 (3.5%)	28 (53.0%)	23 (43.5%)	53 (100.0%)
University clinic	0 (0.0%)	8 (50.0%)	8 (50.0%)	16 (100.0%)
Orthodontic practice (associate)	5 (3.5%)	36 (26.5%)	95 (70.0%)	136 (100.0%)
Total	11 (3.0%)	120 (28.0%)	297 (69.0%)	428 (100.0%)
